# Zn Complex of Diaminedithiol Tetradentate Ligand as a Stable Precursor for ^99m^Tc-Labeled Compounds

**DOI:** 10.3390/molecules25020254

**Published:** 2020-01-08

**Authors:** Shinobu Oshikiri, Tomoya Uehara, Hiroyuki Suzuki, Miho Koike-Satake, Akihiro Hino, Yasushi Arano

**Affiliations:** 1 RI Research Department, FUJIFILM Toyama Chemical Co., Ltd., Chiba 289-1592, Japan; miho.satake@fujifilm.com (M.K.-S.); akihiro.hino@fujifilm.com (A.H.); 2 Laboratory of Molecular Imaging and Radiotherapy, Chiba University, Chiba 260-8675, Japan; tuehara@chiba-u.jp (T.U.); h.suzuki@chiba-u.jp (H.S.); arano@chiba-u.jp (Y.A.)

**Keywords:** ^99m^Tc, radiopharmaceutical, Zn complex, diaminedithiol, N_2_S_2_

## Abstract

The diaminedithiol (N_2_S_2_) tetradentate ligand constitutes a useful chelating molecule for preparing ^99m^Tc-labeled compounds of high in vivo stability in high radiochemical yields. However, since the thiol groups in the N_2_S_2_ ligand are easy to be oxidized to disulfide bonds, they need to be protected with an appropriate protecting group, which hinders the broad applications of the N_2_S_2_ ligand for radiopharmaceuticals. In this study, a Zn chelate of N_2_S_2_ was evaluated as a precursor for purification-free ^99m^Tc-labeled N_2_S_2_ under the mild and simple procedure. Zn-N_2_S_2_ was prepared by reacting Zn acetate with N_2_S_2_, and the Zn-N_2_S_2_ remained stable under aerobic conditions at room temperature. ^99m^Tc-N_2_S_2_ was obtained over 90% radiochemical yields at room temperature by a one-pot reaction, consisting of Zn-N_2_S_2_ (10^−5^ M), ^99m^TcO_4_^−^, ethylenediaminetetraacetic acid (EDTA), and a reducing agent (Sn^2+^) at pH = 5.5 to 7.5. ^99m^Tc-N_2_S_2_ was also obtained over 90% radiochemical yields when the reaction was conducted in the presence of an equimolar amount of IgG antibody. These findings indicate the Zn complex of N_2_S_2_ ligand constitutes a stable and useful precursor to prepare ^99m^Tc-labeled N_2_S_2_ compounds in high yields under the mild and simple procedure.

## 1. Introduction

The growth and broad applications of diagnostic nuclear medicine have been mainly driven by the artificial radionuclide, technetium-99m (^99m^Tc), due to its availability from a portable ^99^Mo-^99m^Tc generator system and its almost ideal physical properties for external imaging. Although recent efforts are being made to develop radiopharmaceuticals derived from positron emitters such as ^11^C and ^18^F, over 70% of diagnostic practices are still conducted with ^99m^Tc. The cost-effectiveness of ^99m^Tc-based radiopharmaceuticals contributes to the medical economy in both developed and developing countries. Thus, ^99m^Tc will continue to be used as one of the essential radionuclides for diagnostic nuclear medicine. Radioactive rhenium (^186^Re and ^188^Re), the congener of Tc, emits beta rays appropriate to targeted radiotherapy to form radiotheranostic pairs with their ^99m^Tc counterparts due to the chemical analogy between Tc and Re. Thus, further development of ^99m^Tc-radiopharmaceuticals constitutes a crucial issue for cost-effective and successful patient management.

**Scheme 1:**
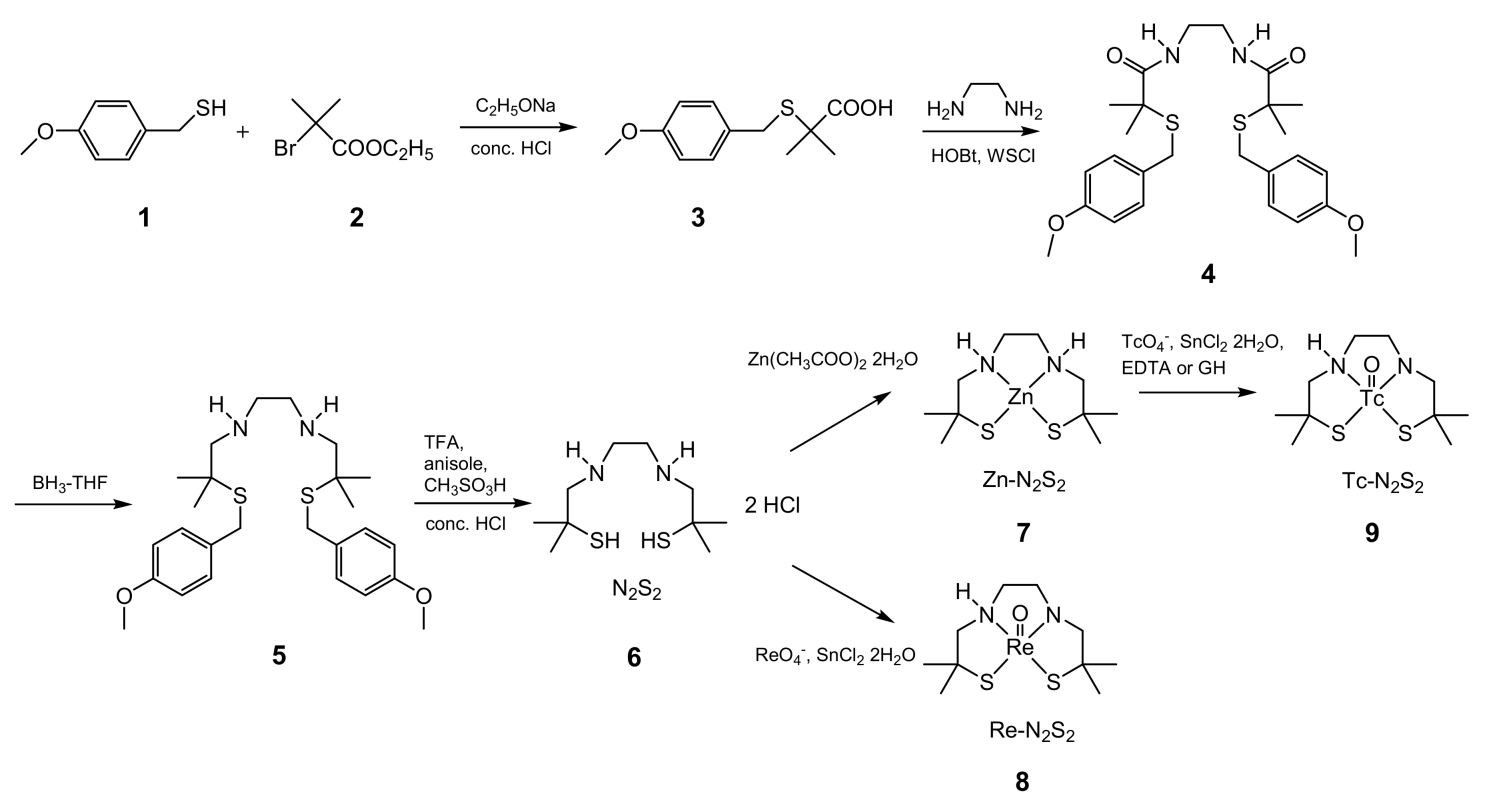
Synthetic procedure for N_2_S_2_ and its Zn, Re, and ^99m^Tc complexes.

A tetradentate chelating agent containing two amines and two thiol groups, referred to as a diaminedithiol (DADT) or a bis(amino ethanethiol: BAT), forms a neutral, lipophilic, and stable complex with the pentavalent oxo-Tc and oxo-Re (TcO^3+^ and ReO^3+^) [1,2]. Such characteristics render DADT attractive as a coordination molecule for ^99m^Tc- and ^186/188^Re-labeled compounds [3,4,5,6]. A presentative structure of DADT ligand is shown in Scheme 1, compound **6** (N_2_S_2_). Indeed, ^99m^Tc-labeled *N,N*′-ethylene bis (l-cysteine ethyl ester) has been used as a radiopharmaceutical for measuring cerebral blood flow [7]. However, further applications of DADT ligands are hindered due to the inherent easily oxidized property of the thiol groups during the storage.

Some acylating agents have been used to protect the thiol groups in N_2_S_2_- or N_3_S_1_-type ligands such as mercaptoacetyl glycyl-glycyl-glycine. These include benzoyl [8], acetyl [9], and *m*-phthalic acid [10]. The deprotection of the benzoyl group is usually conducted at an elevated temperature (e.g., in boiling water) under alkaline conditions (pH > 10) [8,11]. The acetyl protecting group can be removed under milder conditions, whereas a long reaction time (1 h) is needed at room temperature [9]. The *m*-phthalic acid protecting group can be removed at room temperature in the presence of 1 M of NH_2_OH [10], which may necessitate post-labeling removal of NH_2_OH before injection to subjects. Thus, a protecting group that provides a precursor of N_2_S_2_ stable against oxidation and generates ^99m^Tc-N_2_S_2_ in high radiochemical yields under mild conditions without post- or pre-labeling purification is highly useful for developing new ^99m^Tc-radiopharmaceuticals.

Meanwhile, a transmetallation reaction between Zn chelate of dithiocarbamates (DTCs) and rhenium tricarbonyl has been reported. In this reaction, the thiol groups in the DTCs were stabilized upon complexation with Zn ion, and the direct reaction of the Zn complex with Re(CO)_3_Br_3_ provided Re(CO)_3_-DTCs in high radiochemical yields [12]. These results suggested that Zn ion would also be applicable as a protecting agent for the thiol groups in N_2_S_2_ ligands removable during the complexation reaction with ^99m^Tc and Re. Indeed, a prior study showed the formation of Zn-N_2_S_2_ chelate [13]. Zn is classified as the least toxic group of all metals by the ICH Harmonized Guideline [14], which is advantageous to clinical applications.

In the present study, the Zn ion was evaluated as a protecting agent for the thiol groups in N_2_S_2_ ligands using compound 6. After preparing the Zn complex of 6, the reaction parameters that affected the radiochemical yields of the ^99m^Tc-N_2_S_2_ were investigated. The formation of ^99m^Tc-N_2_S_2_ from Zn-N_2_S_2_ was also evaluated in the presence of an equimolar amount of IgG antibody to estimate the applicability to Zn-N_2_S_2_ as a chelating moiety of a bifunctional chelating agent for ^99m^Tc-labeled polypeptides.

## 2. Results

### 2.1. Synthesis

The N_2_S_2_ ligand 6 was synthesized according to the procedure of Ohmomo et al. as shown in Scheme 1. 4-(Methoxyphyenyl)methanethiol 1 was reacted with ethyl 2-bromo-2-methyl propanoate 2 to prepare 3, followed by the condensation with ethylenediamine to provide 4. After reducing the amide bonds in 4 with BH_3_, the thiol protecting group in the resulting compound 5 was removed with TFA/anisole/methanesulfonic acid to obtain 6.

The Zn complex of the N_2_S_2_ was prepared by mixing Zn acetate with compound 6 in an aqueous solution at neutral pH. The Zn-N_2_S_2_ was obtained by extracting the reaction solution with dichloromethane, followed by recrystallization from the mixture of chloroform and hexane in 89% yield. The Zn-N_2_S_2_ remained stable at room temperature under aerobic conditions over several days.

The non-radioactive ^185/187^Re-N_2_S_2_ complex was synthesized by reacting 6 with ReO_4_^−^ in aqueous ethanol using Sn^2+^ as a reducing agent. After silica-gel column chromatography, the oxorhenium complex of N_2_S_2_ was obtained in ca. 20% yield. The structure of this complex was confirmed by mass spectrometry (MS) and infrared spectroscopy (IR).

### 2.2. ^99m^Tc Complexation Reaction

**Figure 1:**
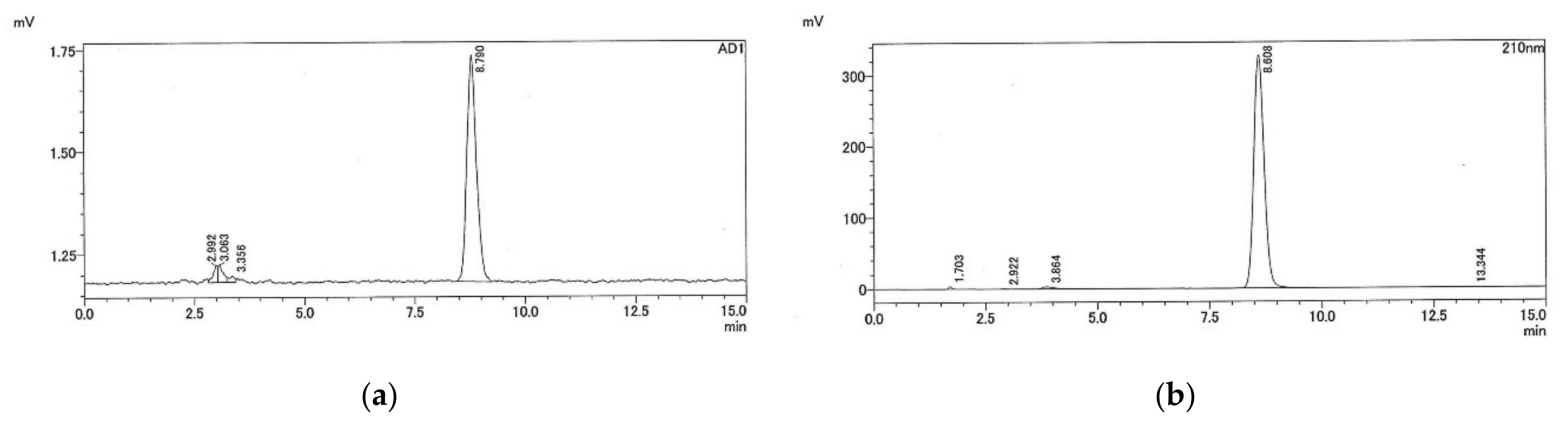
Reversed-phase high-performance liquid chromatography (HPLC) radio-ultraviolet (UV) chromatograms: (**a**) ^99m^Tc-N_2_S_2_; (**b**) Re-N_2_S_2_.

**Figure 2:**
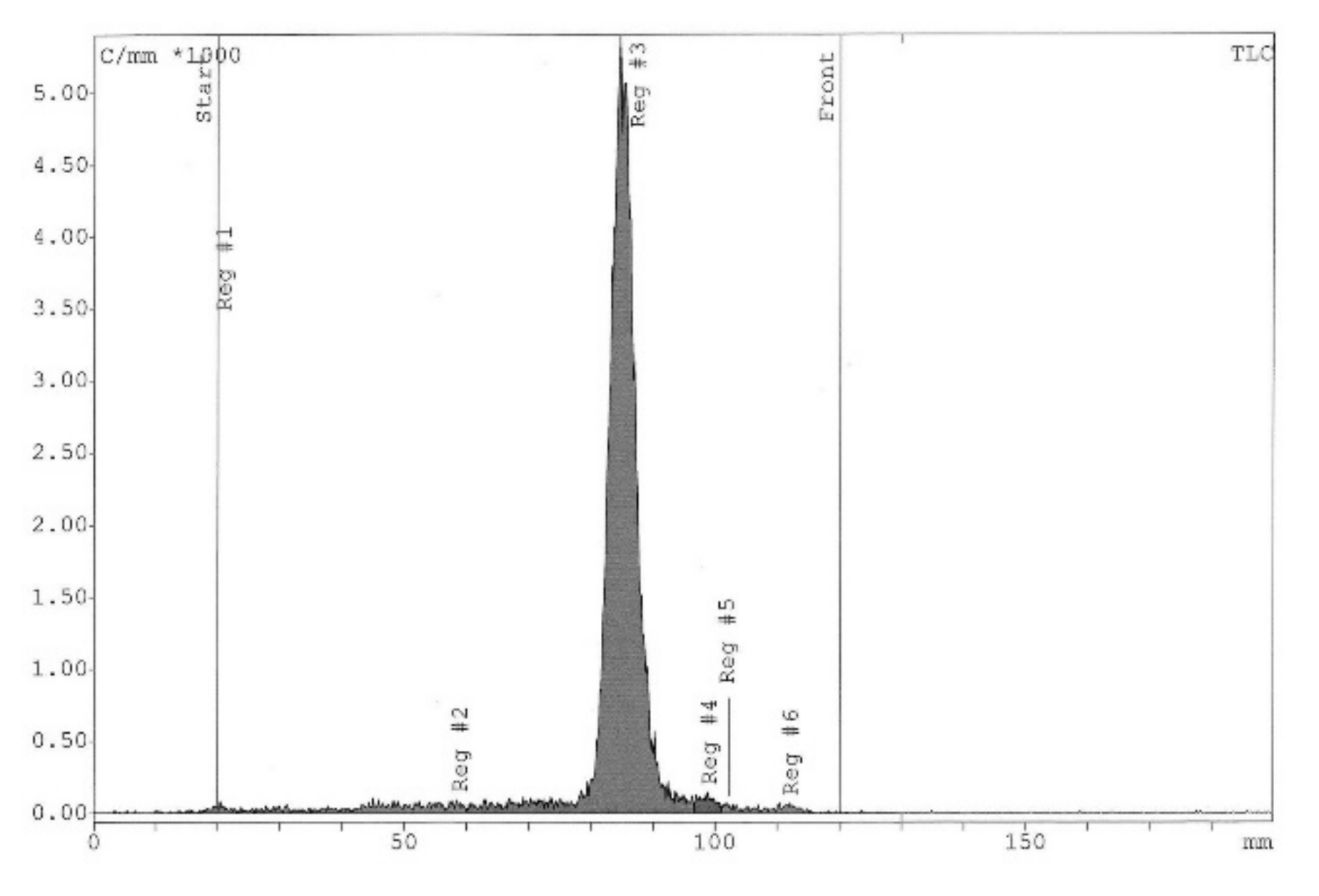
Thin-layer radiochromatography (TLC) of ^99m^Tc-N_2_S_2._

The reaction of Zn-N_2_S_2_ with ^99m^Tc was evaluated at room temperature for future applications as a bifunctional chelating agent for labeling heat-sensitive polypeptides. The reaction parameters for preparing ^99m^Tc-N_2_S_2_ from Zn-N_2_S_2_, which included the reaction pH, Zn-N_2_S_2_ concentration, presence or absence of ethylenediaminetetraacetic acid (EDTA) or glucoheptonate (GH), and the reaction time, were considered. Figure 1 and Figure 2 show typical reversed-phase high-performance liquid chromatography (RP-HPLC) and thin-layer radiochromatography (TLC) of ^99m^Tc-N_2_S_2_ and Re-N_2_S_2_. The RP-HPLC retention time of ^99m^Tc-N_2_S_2_ (8.8 min) was similar to that of ^185/187^Re-N_2_S_2_ (8.6 min) verified by MS, IR, elemental analysis, and proton nuclear magnetic resonance (^1^H-NMR). These results, along with previous studies [15,16], supported that the ^99m^Tc-N_2_S_2_ would possess the chemical structure shown in Scheme 1.

**Figure 3:**
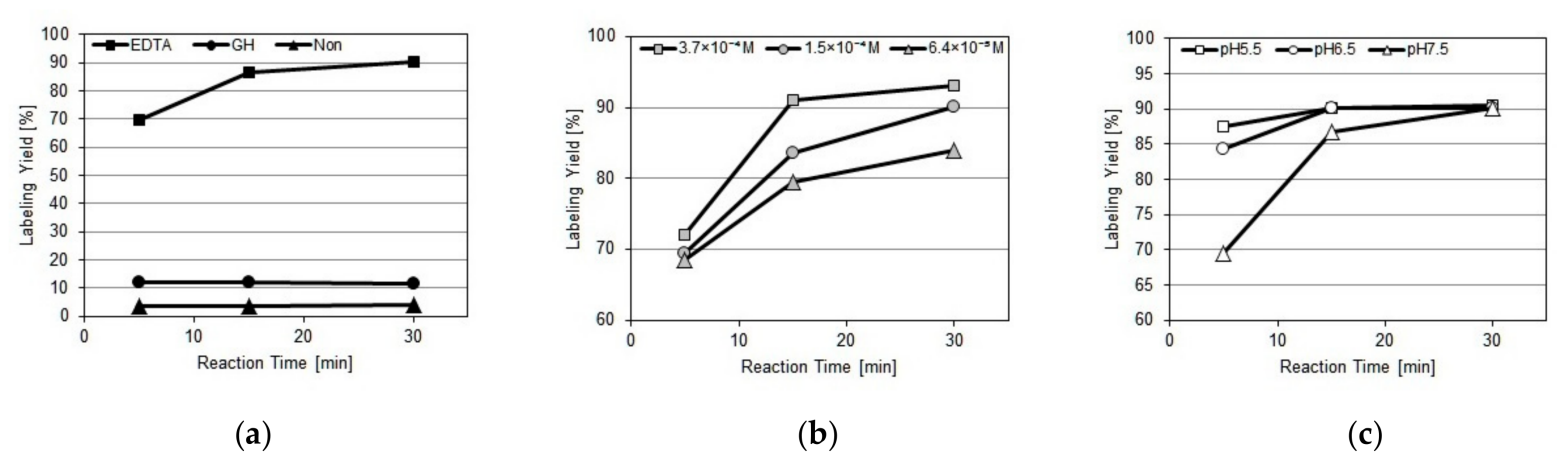
The radiochemical yields of ^99m^Tc-N_2_S_2_ as a function of (**a**) ethylenediaminetetraacetic acid (EDTA) or glucoheptonate (GH) (pH = 7.5, Zn-N_2_S_2_: 1.0 × 10^−5^ M, EDTA: 1.5 × 10^−4^ M, GH: 1.5 × 10^−4^ M); (**b**) EDTA concentration (pH = 7.5, Zn-N_2_S_2_: 1.0 × 10^−5^ M); (**c**) reaction pH (Zn-N_2_S_2_: 1.0 × 10^−5^ M, EDTA: 1.5 × 10^−4^ M).

Figure 3 shows the results from a preliminary experiment as a function of reaction parameters. ^99m^Tc-N_2_S_2_ was obtained in high radiochemical yields only when the reaction was conducted in the presence of EDTA. The reaction did not proceed without EDTA, while the presence of GH in place of EDTA resulted in low radiochemical yields (Figure 3a). The increase in EDTA concentration in the reaction mixture increased the radiochemical yields of ^99m^Tc-N_2_S_2_ (Figure 3b). The radiochemical yields of ^99m^Tc-N_2_S_2_ decreased as the reaction pH increased from pH = 5.5 to 7.5. However, the radiochemical yields reached similar to one another at 30 min (Figure 3c).

**Figure 4:**
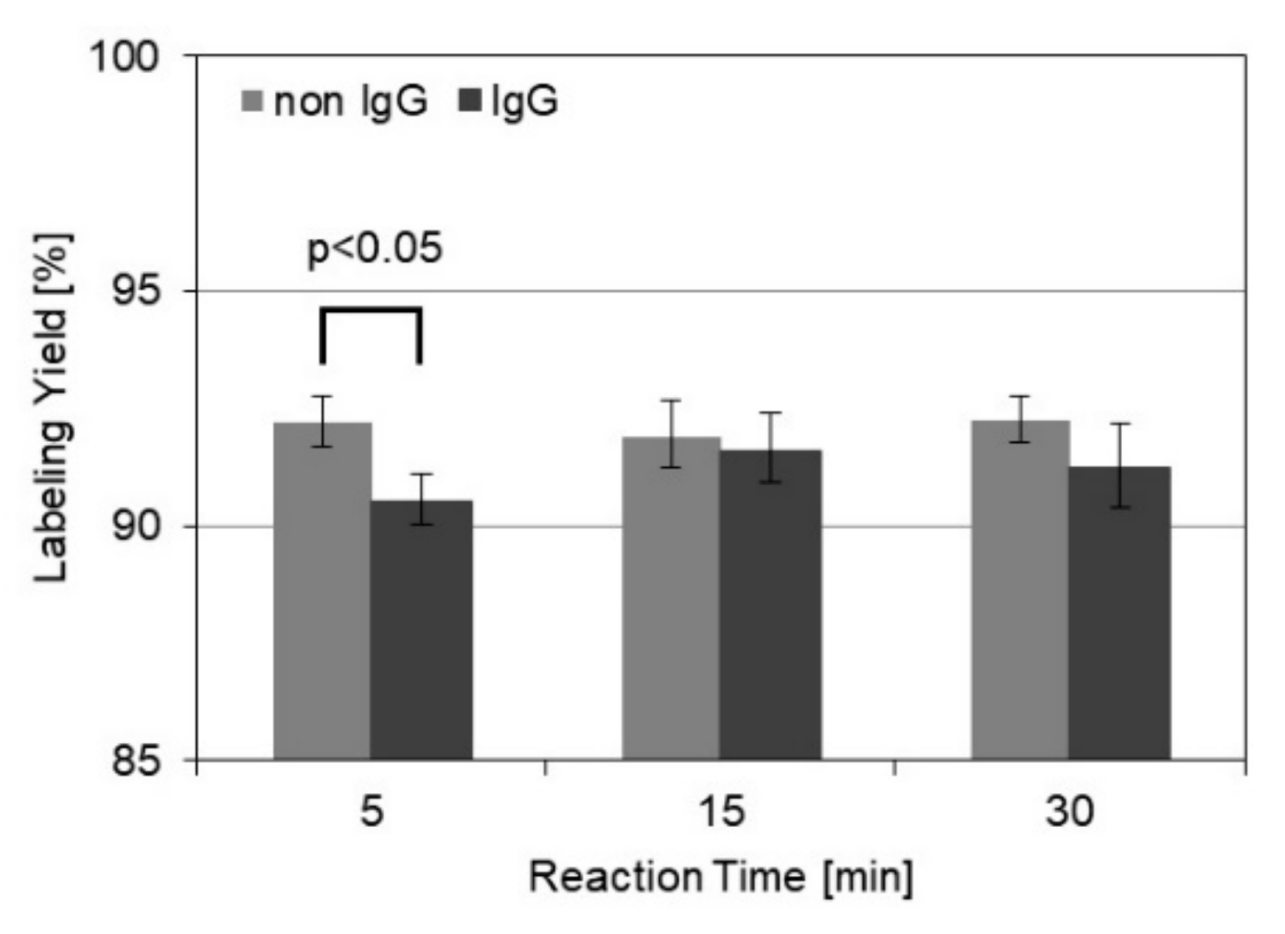
The radiochemical yields of ^99m^Tc-N_2_S_2_ in the presence or absence of an equimolar amount of IgG (EDTA: 1.5 × 10^−4^ M, pH = 5.5, Zn-N_2_S_2_: 1.0 × 10^−5^ M, IgG: 0, or 1.0 × 10^−5^ M).

Figure 4 shows the radiochemical yields of ^99m^Tc-N_2_S_2_ under the optimal conditions in the presence or absence of an IgG equimolar amount to that of Zn-N_2_S_2_. No significant differences were observed in the radiochemical yields of ^99m^Tc-N_2_S_2_ between the two experimental conditions.

## 3. Discussion

^99m^Tc radiopharmaceuticals are usually prepared under sterile conditions by mixing a solution of ^99m^TcO_4_^−^with a kit formulation consisting of a ligand and a reducing agent. The in situ deprotection of N_2_S_2_ ligand and subsequent ^99m^Tc complexation reaction is preferable for clinical applications. In this study, the one-pot synthesis of ^99m^Tc-N_2_S_2_ from Zn-N_2_S_2_ was investigated at room temperature for the applications to heat-sensitive molecules. The Zn-N_2_S_2_ concentration of 1.0 × 10^−5^ M was selected for future applications to imaging the saturable systems of the body [16].

Since the direct reaction of ^99m^TcO_4_^−^ and Zn-N_2_S_2_ in the presence of a reducing agent, Sn^2+^, failed to produce ^99m^Tc-N_2_S_2_ (Figure 3a), GH was added to the reaction mixture to stabilize the pentavalent ^99m^TcO^3+^ against hydrolysis [16]. Under the conditions, small amounts of ^99m^Tc-N_2_S_2_ (ca. 10%) were obtained with unchanged radiochemical yields with the reaction time (Figure 3a). These results suggested that GH might rather act as a weak demetallation agent to produce N_2_S_2_ from Zn-N_2_S_2_. The concentration of GH was 15-times higher than that of Zn-N_2_S_2_. The stability constant for Zn-GH was assumed to be close to the stability constant for Zn-gluconic acid (1.70) [17], considering the similar chemical structures and acid deprotonation constants between GH and gluconic acid [18]. Thus, a deprotection agent of higher stability constant with Zn ion was then investigated to assess the working hypothesis.

To facilitate the removal of Zn ion from Zn-N_2_S_2_, EDTA was selected due to its high stability constant of 16.44 with Zn ion [19]. As shown in Figure 3a, over 90% radiochemical yields were achieved under the Zn-N_2_S_2_ concentration of 10^−5^ M at room temperature for 30 min. The radiochemical yields of ^99m^Tc-N_2_S_2_ increased with an increase in the EDTA concentration (Figure 3b), indicating that the demetallation of Zn from Zn-N_2_S_2_ constituted the rate-determining step for the synthesis of ^99m^Tc-N_2_S_2_ from Zn-N_2_S_2_. It should be noted that EDTA also forms a complex with the trivalent Tc [20,21]. However, since much higher EDTA concentrations (ca. >10^−3^ M) are needed to prepare ^99m^Tc-EDTA [20,21], EDTA acted as a demetallation agent to generate N_2_S_2_ from Zn-N_2_S_2_ under the present reaction conditions. These results also indicated that N_2_S_2_ preferentially provided its ^99m^Tc complex in high radiochemical yields under the presence of higher concentrations of labile chelating molecules. Indeed, ^99m^Tc-N_2_S_2_ was obtained in high radiochemical yields in the presence of an equimolar amount of IgG, as shown in Figure 4. The formation of ^99m^Tc-N_2_S_2_ under a wide range of reaction pH rendered Zn-N_2_S_2_ applicable to a variety of biomolecules of interest (Figure 3c). The gathered findings indicate that the Zn-N_2_S_2_ constitutes a useful precursor to prepare a variety of ^99m^Tc-N_2_S_2_-based radiopharmaceuticals at low Zn-N_2_S_2_ concentrations under mild reaction conditions by a simple procedure.

## 4. Materials and Methods

### 4.1. Materials

All chemicals and an antibody were reagent grade and used without further purification. The pertechnetate-99m solution was obtained from a commercial ^99^Mo-^99m^Tc Generator (Ultra-Techne Kow, FUJIFILM Toyama Chemical Co., Ltd., Tokyo, Japan).

### 4.2. Equipment

Radiochemical purities were determined with a Radio-Thin Layer Chromatography (TLC) Analyzer (GITA-STAR, Elysia-RAYTEST, Straubenhardt, Germany). High-performance liquid chromatography (HPLC) analyses were performed using a SHIMADZU model LC-20AD (Kyoto, Japan).

### 4.3. Syntheses

The N_2_S_2_ ligand, 1,1′-(ethane-1,2-diylbis(azanediyl))bis(2-methylpropane-2-thiol) dihydro-chloride, was synthesized according to the procedure of Ohmomo et al. [22].

Zn complex of N_2_S_2_ ligand: Under an argon atmosphere, 1,1′-(ethane-1,2-diylbis-(azanediyl))bis(2-methylpropane-2-thiol) dihydrochloride 50.0 mg (0.162 mmol) and anhydrous sodium acetate 19.1 mg (0.233 mmol) were dissolved in H_2_O (1.3 mL) at room temperature. A mixture of Zn(II) aceteate dehydrate 59.1 mg (0.269 mmol) and anhydrous sodium acetate 19.0 mg (0.232 mmol) in 1.3 mL of H_2_O was added dropwise to the solution. The reaction mixture was stirred at room temperature for 1.5 h. The solution was extracted with 10 mL of dichloromethane three times. The organic solution was dried over Na_2_SO_4_. After filtration, the filtrate was evaporated, and the residue was recrystallized from chloroform and hexane to afford Zn-N_2_S_2_ as a white powder. Yield 43.4 mg (89.4%). ESI-MS, C_1__0_H_22_N_2_S_2_Zn [M + H]^+^ *m*/*z* 299.06, found: 299.11. Anal. C_10_H_22_N_2_S_2_Zn·0.6Na_2_SO_4_: C, 31.19; H, 5.76; N, 7.28%, found: C, 31.59; H, 5.75; N, 7.09%.

Oxorhenium(V) complex of N_2_S_2_ ligand: The N_2_S_2_ ligand 62.3 mg (2.01 mmol) in 7 mL of 50% (*v*/*v*) ethanol/water was added to a solution of potassium perrhenate 58.2 mg (0.201 mmol) in 30 mL of 50% (*v*/*v*) ethanol/water. Tin(II) chloride dehydrate 45.5 mg (0.202 mmol) in 7 mL of 50% (*v*/*v*) ethanol/water was then added to the mixture, and the reaction mixture was stirred at room temperature for 2 h. After removing ethanol by evaporation, an aqueous solution of 1 M potassium carbonate was added to bring the reaction pH neutral. The solution was extracted with 100 mL of ethyl acetate three times. The organic solution was dried over Na_2_SO_4_. After filtration, the filtrate was evaporated to dryness. The residue was purified by silica gel chromatography eluted with a mixture of dichloromethane and methanol (95:5) to provide a purple crystal of ReO-N_2_S_2_. Yield 16.8 mg (19.2%). ESI-MS, Mass C_1__0_H_21_N_2_OReS_2_ [M + H]^+^ *m*/*z* 437.07, found 437.19. Anal. C_10_H_21_N_2_OReS_2_: C, 27.57; H, 4.86; N, 6.43%, found: C, 27.34; H, 4.73; N, 6.22%. IR (KBr) 920 cm^−1^ (Re=O). ^1^H-NMR (400 MHz, CD_3_OD) δ1.46 (s, 6H), 1.60 (s, 3H), 1.80 (s, 3H), 2.99 (br, 2H), 3.37 (br, 2H), 3.76 (br, 4H).

### 4.4. Technetuim-99m Complexatoin Reaction

All the solutions were bubbled with a stream of N_2_ before use. A 1-mL solution of pertechnetate-99m (^99m^TcO_4_^−^, 581 ± 185 MBq) was added to 0.5 mL of 0.2 M of sodium phosphate buffer (pH = 5.5, 6.5 and 7.5) and 0.1 mL of Zn-N_2_S_2_ (6.39, 38.3 and 230 μg) ethanol solution. Then, 0.5 mL of SnCl_2_·2H_2_O (2.1 × 10^−^^4^ M) solution or SnCl_2_·2H_2_O (2.1 × 10^−^^4^ M) containing a transfer ligand (EDTA·2Na·2H_2_O 6.4 × 10^−^^4^ M or GH sodium salt 6.4 × 10^−^^4^ M) aqueous solution was added to the mixture. The mixture was stood at room temperature for 5, 15, and 30 min. The effect of EDTA concentrations (1.0, 6.1, and 37 × 10^−5^ M) on the radiochemical yields was also evaluated.

The radiochemical yields of ^99m^Tc-N_2_S_2_ were also determined in the presence of an equimolar amount of IgG at pH = 5.5 in the presence of 1.5 × 10^−4^ M of EDTA. The concentration of Zn-N_2_S_2_ and the IgG were 1.0 × 10^−5^ M.

### 4.5. Measurement of Radiochemical Yields of ^99m^Tc-N_2_S_2_

Radiochemical yields of ^99m^Tc-N_2_S_2_ were determined by TLC method with the C18 reversed-phase TLC plate (NAGEL RP-18W/UV254) eluted with a mixture of acetone and 0.5 M of ammonium acetate (65:35). Under the conditions, ^99m^Tc-N_2_S_2_ had a Rf value of 0.6–0.7, while the Rf values of ^99m^TcO_4_^−^ and ^99m^TcO_2__._nH_2_O were 0.9 to 1.0 and 0, respectively.

### 4.6. Characterization of ^99m^Tc-N_2_S_2_ and Re-N_2_S_2_

The HPLC retention time of ^99m^Tc-N_2_S_2_ and Re-N_2_S_2_ was compared with SHISEIDO CAPCELL PAK UG120 (5 μm, 4.6 × 150 mm) eluted with 45% (*v*/*v*) aqueous methanol at 40 °C under a flow rate of 1 mL/min.

### 4.7. Statistical Analysis

Results were statistically analyzed using EXSUS Version 8.1. Differences were considered statistically significant when *p* was < 0.05. A Shapiro–Wilk test was used to determine normality. When data were normally distributed for two groups, a student’s *t*-test was used. If that was not the case, the nonparametric Wilcoxon’s rank sum test was used.

## 5. Conclusions

The Zn-N_2_S_2_ was easily synthesized, remained stable under aerobic conditions, and provided ^99m^Tc-N_2_S_2_ in high radiochemical yields under a mild one-pot reaction at the Zn-N_2_S_2_ concentration of 10^−5^ M. The formation of ^99m^Tc-N_2_S_2_ was not hindered by the presence of labile chelators, such as EDTA and an IgG antibody. The gathered findings would facilitate the development of cost-effective kit formulations for ^99m^Tc-radiopharmaceuticals using the N_2_S_2_ ligand as the chelating moiety.
